# Performance characteristics of Fungitell STAT™, a rapid (1→3)-β-D-glucan single patient sample *in vitro* diagnostic assay

**DOI:** 10.1093/mmy/myaa028

**Published:** 2020-05-13

**Authors:** Robert L D'Ordine, Kevin A Garcia, Josee Roy, Yonglong Zhang, Barbara Markley, Malcolm A Finkelman

**Affiliations:** Associates of Cape Cod, Inc., East Falmouth, Massachusetts, USA

**Keywords:** fungal, infection, diagnosis, (1→3)-β-glucan, method

## Abstract

Serum (1→3)-β-D-glucan (BDG), is an adjunct test in the diagnosis of invasive fungal disease (IFD). Fungitell STAT™, a facile, rapid, single patient option, executable for one or more patient specimens in approximately an hour, has been developed to address a need for rapid in-house testing. This method presents qualitative information concerning serum BDG levels, using an index value that allows the rapid categorization of patients as positive, negative, or indeterminate relative to serum BDG titer. The categorical and analytical performance of Fungitell STAT was evaluated. The categorical agreement between methods was established by testing patient samples which had been previously categorized with Fungitell. Receiver Operating Characteristic curves were used to identify cut-offs using 93 de-identified patient specimens. Subsequently, using these cutoffs, an independent group of 488 patient specimens was analyzed. Positive percent agreement (PPA) with, and without, indeterminate results was 74% and 99%, respectively. Negative percent agreement (NPA) was 91% and 98% with, and without, indeterminate results, respectively. Additionally, commercially available normal off-the-clot sera were spiked with *Saccharomyces cerevisiae*-derived (1→3)-β-D-glucan to produce analytical samples. Analytical reproducibility using spiked samples was excellent with 94% of the CV (coefficient of variation) values ≤10% among three independent laboratories. Good correlation with the predicate method was demonstrated with correlation coefficients of 0.90 or better with patient samples and 0.99 with spiked samples. The Fungitell STAT index assay provides a rapid and suitable method for serum BDG testing.

## Introduction

Invasive fungal disease (IFD) is a condition characterized by high morbidity and mortality and is diagnostically challenging.^1–3^ Delay in diagnosis can have severe consequences given that as little as 12–24 hours of delay in appropriate therapy has been demonstrated to dramatically worsen mortality.^4^ Importantly, timely diagnosis and appropriate therapy of IFD has been widely demonstrated to produce significant reductions in mortality.^1,5,6^ The mainstay of IFD diagnosis, fungal culture, is considered the gold standard yet is repeatedly shown to be insensitive and can require days to obtain actionable information.^7–10^ Newer diagnostic modalities have been developed that offer rapid, near point of care testing (NPOCT) for IFD with improved performance including, importantly, very high negative predictive value (NPV).^11,12^ These offer the potential to reduce the widespread, inappropriate empiric administration of systemic antifungals, thus supporting antimicrobial stewardship goals.^13–16^

(1→3)-β-D-glucan is a cell wall polysaccharide found in nearly all fungal species with the exception of the Mucorales.^17,18^ Fungitell^®^, a nearly panfungal serum (1→3)-β-D-glucan (BDG) assay, has been available for nearly two decades as an adjunct test in the diagnosis of IFD.^19^ While available as a rapid microplate-based test, it is designed for batch testing of up to 21 patients.^20^ This multipatient capability serves large institutions and reference laboratories with high sample numbers well. However, a lower throughput option would be preferred in emergent clinical settings, which often only require one or a few tests at a time. To meet this need, Fungitell STAT™ was developed as an adaptation of the original kit and is suitable for testing one or more patient specimens in approximately an hour. Fungitell STAT™ presents qualitative information concerning patient serum BDG levels, using an index value result format that is both familiar to the infectious disease community and which allows the rapid stratification of patients as diagnostically positive, negative, or indeterminate relative to serum BDG burden. Here we describe results demonstrating the comparability of the diagnostic performance of the Fungitell STAT™ relative to its predicate device, Fungitell^®^.

## Methods

### Serum specimens

Two types of serum samples were evaluated. Specimens submitted for clinical laboratory testing, from patients suspected of invasive fungal infection, were fully de-identified and used as clinical samples in these studies. The de-identification performed met the requirements of the US Department of Health and Human Services Office of Human Research Protection and eliminated any possibility of patient identification. In addition, commercially available normal off-the-clot sera were spiked with *Saccharomyces cerevisiae*-derived (1→3)-β-glucan (SCBG). Both sample types were utilized for testing with Fungitell^®^ and Fungitell STAT™.

### Reagents

Fungitell^®^ kits (Associates of Cape Cod, Inc., Falmouth, MA, USA) were used to determine serum BDG titers with output in pg/mL. Serum samples were processed according to the manufacturer's instructions for use.

Fungitell STAT™ (Associates of Cape Cod, Inc., Falmouth, MA, USA) uses the following:

1. Fungitell STAT™ reagent tubes: Individual tubes (12 × 65 mm borosilicate, depyrogenated) containing lyophilized BDG-specific reagent sufficient for one sample.
2. Fungitell STAT™ Standard: Individual tubes (as described above) containing lyophilized BDG equivalent to the Fungitell^®^ positive cutoff level of 80 pg/mL ± 8 pg/mL when reconstituted with the appropriate lot-specific volume of water.
3. BDG-free water.
4. Alkaline pretreatment solution (APS): 0.6 M KCl/0.125 M KOH.

The Fungitell STAT™ Reagent used in these studies contains the same *Limulus* Amebocyte lysate active components as in the predicate device Fungitell^®^.

The SCBG (Saccharomyces cerevisiae beta-glucan) used in the Fungitell STAT™ Standard in these studies is a (1→3)-β-D-glucan hot water cell wall extract of baker's yeast. The SCBG is filtered, autoclaved, and stored at −80°C until use. The SCBG is diluted, and concentration is assessed against the Fungitell^®^ predicate device Glucan Standard.

### Fungitell^®^ reaction method

Briefly, 5 *µ*L of serum are treated with 20 *µ*L of alkaline pretreatment solution (0.125 M KOH/0.6 M KCl) [1:4 ratio of serum to pre-treatment solution] and incubated at 37°C ± 1°C for 10 minutes. Subsequently, 100 *µ*L of reconstituted Fungitell^®^ reagent [1:4 ratio of reaction sample to reagent] are added to the sample wells for kinetic reaction monitoring at 405 and 495 nm. The Vmean (milliabsorbance units/min) of the A405 nm minus A490 nm for the samples are interpolated against a similarly constructed standard curve. The output for the standards and the samples is in picograms/ml (pg/mL).

### Fungitell STAT™ reaction method

Briefly, 75 *µ*L or 50 *µ*L of serum are treated, in 12 × 75 mm, depyrogenated borosilicate tubes, with 300 *µ*L or 200 *µ*L (1:4 ratio is constant), respectively, with APS (0.125 M KOH/0.6 M KCl) and incubated at 37°C ± 1°C for 10 minutes. Subsequently, 75 *µ*L of the serum-APS solution(s) are added to a reagent tube containing Fungitell^®^ reagent reconstituted in 300 *µ*L of BDG-free water for kinetic reaction monitoring. Fungitell STAT™ standards are reconstituted with a specified, lot-specific amount of BDG-free water and APS, for example, 95 *µ*L and 380 *µ*L, respectively, and further processed in the same manner as serum samples. For efficiency during the method comparison, typically, samples were run in an eight reaction grouping: one Fungitell STAT standard plus seven serum samples. However, one standard and fewer samples were also run during some studies depending on design.

### Instrumentation and software

Fungitell^®^ assays were read using ELx808iu incubating plate readers with Gen 5 v. 2.00.18 software (BioTek, Winooskie, VT, USA) following the instructions for use with the kit. Fungitell STAT™ assays were read using a PKF08 incubating tube reader (Lab Kinetix, Hutto, TX, USA), capable of reading optical density at 405 and 495 nanometers. Data acquisition and processing utilized Beta Glucan Analytics software v. 1.0 (Associates of Cape Cod, Inc., Falmouth, MA, USA). All reaction mixtures were incubated at 37°C ± 1°C and read kinetically for 40 minutes. The kit configuration permits the testing of between five and eight samples per kit, using the suggested eight well instrument: Five tests comprising one control plus one patient sample or two tests comprising (a) one control plus seven patient samples and (b) one control plus one patient sample.

### External laboratories

Testing was performed at the Associates of Cape Cod, Inc., research laboratories and at three commercial CLIA-certified diagnostic laboratories in Massachusetts. One laboratory was Beacon Diagnostics Laboratory, a division of ACC. The other two laboratories, Lab A and Lab B, are independent laboratories. Prior to testing, the participating laboratory technologists were provided training and were required to demonstrate proficiency with Fungitell STAT™.

### Sample analysis procedures

Spiked serum samples were utilized for all Fungitell STAT™ interlaboratory precision studies. Five (5) samples were spiked with SCBG over the range of the predicate device, spanning all interpretative zones (two negative samples, one indeterminate sample, and two positive samples). These were distributed to all three participating laboratories for intra- and interlab precision analyses. The data were collected by two analysts using two instruments within each participating laboratory.

Patient samples were used for cutoff determination studies (2 analysts, 2 instruments) and method comparison (3 analysts, 3 instruments).

### Data collection and reduction

Fungitell STAT™ data were collected at 37°C in the tube reader. The 405 and 495 nanometer optical densities of Fungitell STAT™ standard and serum sample reaction mixtures were measured at a minimum of 5-second intervals for a period of 40 minutes (2400 seconds). The OD405 minus OD495 value was computed, and the slopes of the line of this data set for the interval between 1900 and 2400 seconds were computed for the Fungitell STAT™standard and the serum samples. A beta-glucan index (BGI) was computed for each sample by dividing the sample slope by the Fungitell STAT™ standard slope (see Table 1). A series of quality control criteria were applied to the observed data in order to confirm the assay performance, and these are included in the instructions for use which can be found in the supplemental file to this paper.

### Statistical analyses

Statistical analyses were performed in Excel 2010 or higher, GraphPad Prism 5.04 or higher and or SAS version SAS V9.3 or higher.

The ROC assessment method followed the guidance document CLSI EP24-A2: “Assessment of the Diagnostic Accuracy of Laboratory Tests Using Receiver Operating Characteristic Curves.” Two cutoff values were identified, one for the negative side and one the positive side, by performing the receiver operating characteristic (ROC) analysis for each side separately (i.e., Fungitell^®^ negative samples vs. Fungitell^®^ indeterminate samples), followed by Fungitell^®^ positive samples vs. Fungitell^®^ indeterminate samples. The data from the test results of the 93 samples falling into the respective zones were parsed and utilized to calculate the sensitivity and 1-specificity necessary to plot the ROC curves below. The calculated sensitivity and 1-specificity was used to calculate Youden index associated with potential STAT index cutoff values for the negative side versus the indeterminate zone and for the positive side versus the indeterminate zone.

Similarly, the percent positive agreement (PPA) and negative percent agreement (NPA) were calculated with and without the indeterminate zone in these analyses as presented in Table 5. They were calculated using the exact (Clopper-Pearson) confidence limits.^21^

## Results

### Assay output example

**Figure 1.**
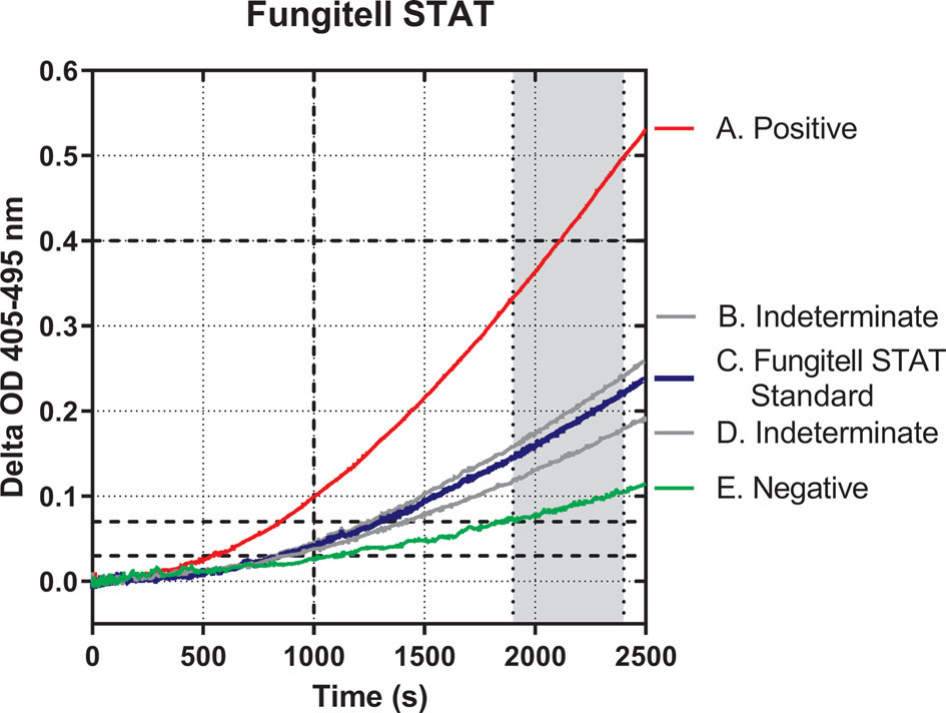
Illustration of kinetic curves derived from the Fungitell STAT™ method. Samples on the graph: (A) is positive, and (B) and (D) are indeterminate. (C) Fungitell STAT™ Standard; (E) negative. All plots are delta OD 405-495 nm. The gray zone between 1900 and 2400 seconds is the area of linear regression from which rates are determined. The dashed lines on the *y*-axis are 0.03, 0.07, and 0.40 delta OD 405-495 nm, respectively. The dashed line on the *x*-axis is at 1000 seconds. These markings relate to the QC criteria described in Supplementary File 1. The reduced data output from these kinetic curves is presented in Table 1.

The Fungitell STAT™ assay data output is represented in graphical form in Figure 1. Reaction mixture kinetic data are presented for positive, indeterminate, and negative serum samples as well as the Fungitell STAT™ standard (see Fig. 1 legend). The reaction kinetic curve shapes reflect those observed for similar samples in the original microplate-based Fungitell^®^ kit. The presence of BDG in the reaction mixture causes the activation of Factor G zymogen serine protease, the BDG-sensitive component of the LAL cascade. This in turn activates pro-clotting enzyme, also a zymogen serine protease, which cleaves para-nitroaniline (pNA) from a tripeptide para-nitroanalide substrate (Boc-Leucine-Glycine-Arginine-pNA) present in the reaction mixture. The appearance of the product pNA is observed at 405 nm. In addition, optical density at 495 nm is also measured, as an indicator of nonspecific optical density changes that may be due to light scattering in some samples. The data plotted in Figure 1 are composed of the OD405-OD495 data set. An initial period of low reactivity (lag phase) is observed, and its duration is dependent upon samples BDG titer. After an inflection indicating activation of the proteolytic cascade, the optical density develops in a mostly linear fashion. The sample index values were computed, as described in the methods (Table 1), by dividing the serum samples’ slope by that of the Fungitell STAT™ standard. This results in a value termed the (1→3)-β-glucan index (BGI).

### Linearity of response

**Figure 2.**
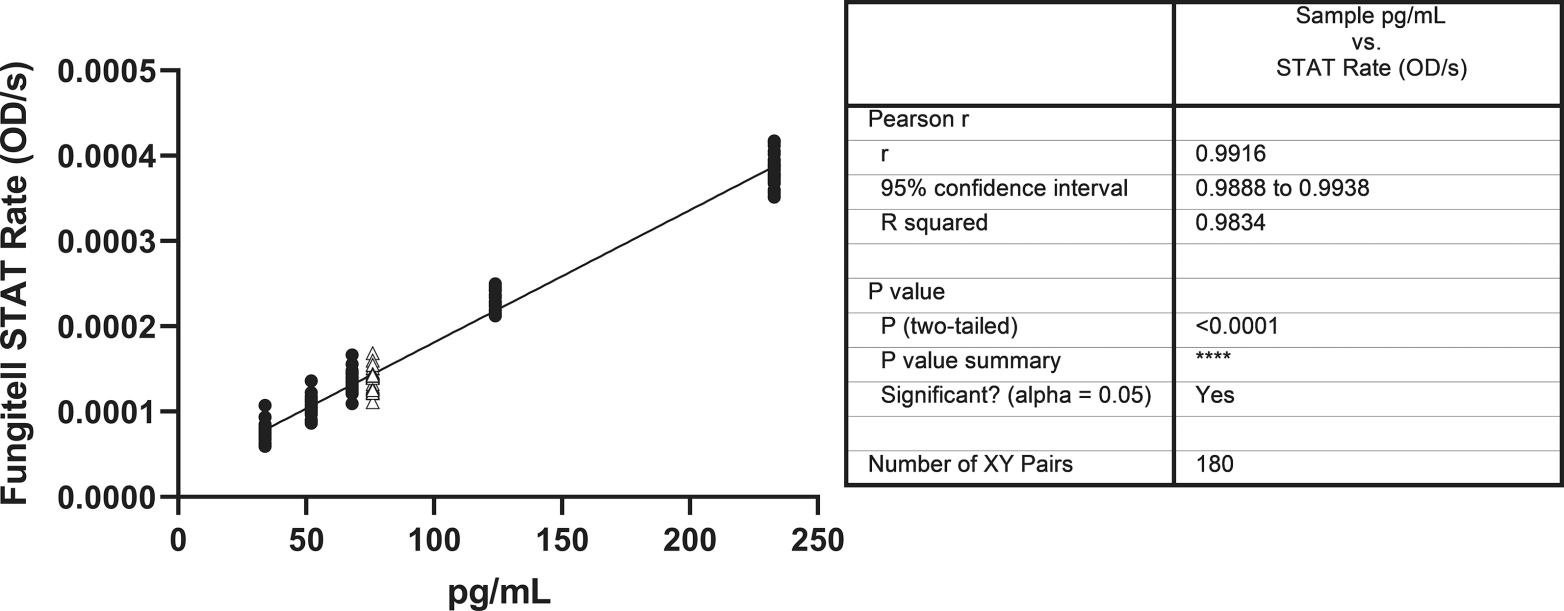
Linearity of response of the Fungitell STAT™ method with spiked serum samples. The y-axis is the Fungitell STAT™ optical density change rate and the *x*-axis is Fungitell^®^ pg/mL. The serum sample concentrations (closed circles) were 34, 52, 68, 124, and 233 pg/mL. The concentration of this Fungitell STAT™ standard (open triangles) is 76 pg/mL when reconstituted in the prescribed volume. Equation of the line: Y = 1.55e-006*X + 2.60e-005, *r*-value = 0.992. There are 180 data points on this plot, 30 data points per each *x*-axis value. These data were collected in an external lab by two analysts using two instruments over five nonconsecutive days, using the same sample set.

**Figure 3.**
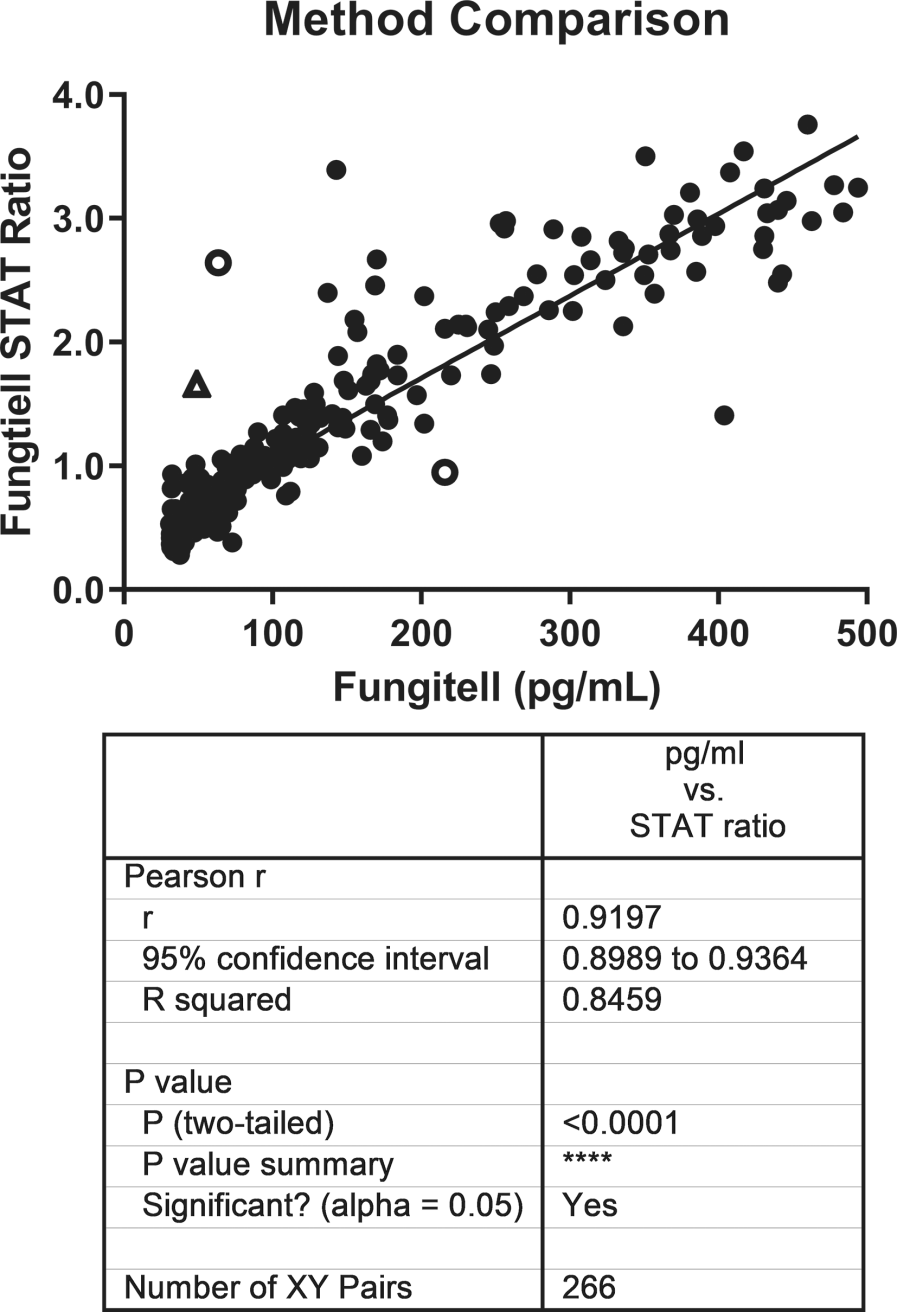
Comparison of Fungitell STAT™ with Fungitell^®^ predicate. The Fungitell STAT™ ratio data and the Fungitell^®^ concentration data for 266 samples were used for this analysis. These 266 samples were those from the population of 488 (see method comparison section) that produced within range, numerical results for both methods. Values that were under- or over-range in either assay were not included in the assessment. Among several discrepant replicates observed three samples of the 266 produced highly discrepant results relative to *both* the correlation and interpretative zones between the two methods. Two of these were indeterminate to positive or positive to indeterminate transitions (open circles). One (open triangle) was a negative to positive transition. This sample was retested and found to be negative as expected. Contamination is suspected in the initial testing of that sample.

The linearity of response of the Fungitell STAT™ method was observed in several different studies. Both patient serum samples and laboratory made serum samples were used in these assessments. Laboratory made serum based spiked glucan samples result in *r*-values that are consistent with the requirements for the predicate device standard curve analytical results with Glucan Standard (*r* value ≥ 0.980), Figure 2. Good linearity was also demonstrated with over 250 unique patient samples in Figure 3. In each case the linear range within the 31 to 500 pg/mL range of the predicate device is characterized by a Pearson *r*-value that is 0.92 or better.

### Determination of positive, negative, and indeterminate zones

**Figure 4.**
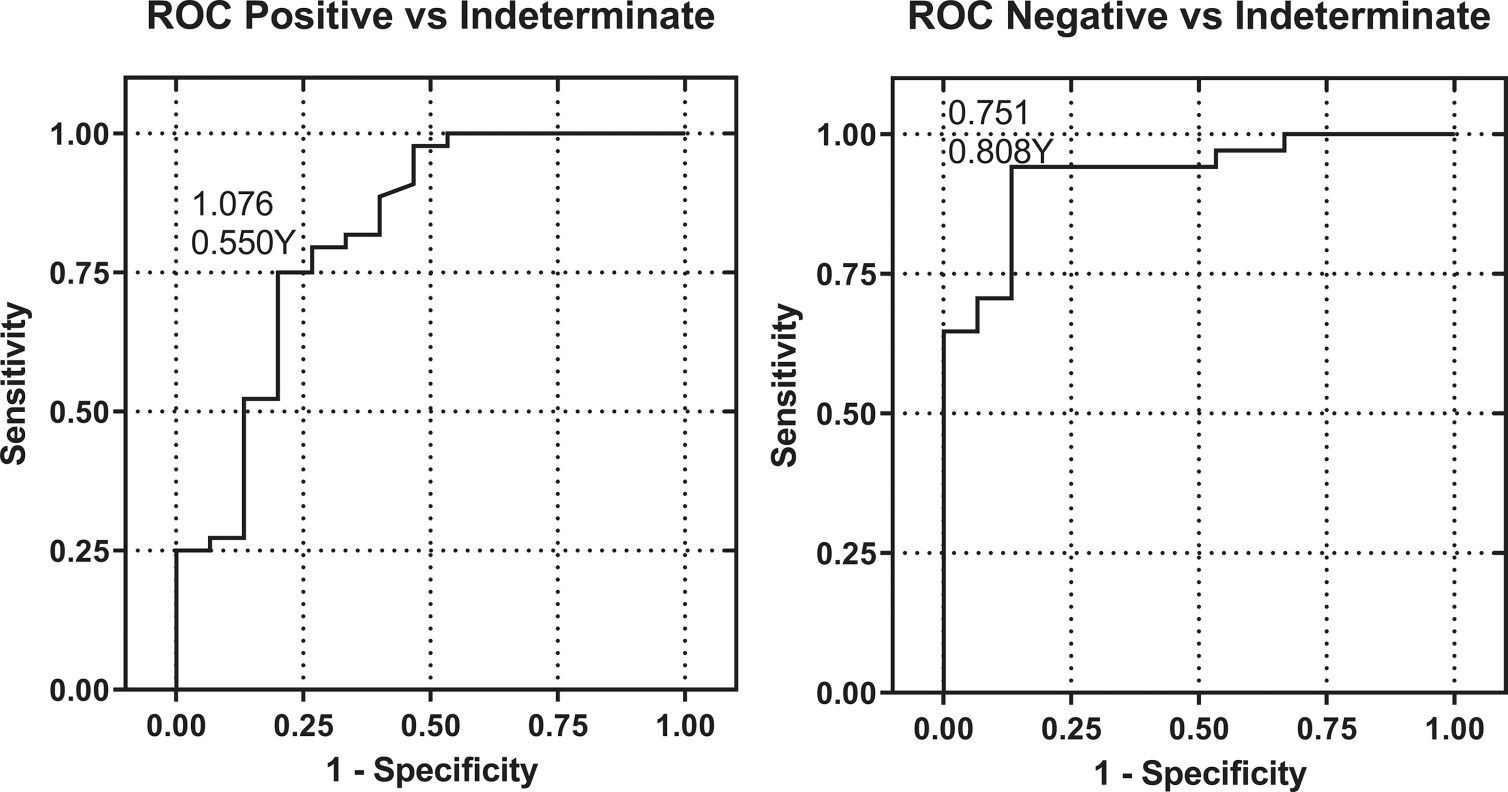
ROC Curves. Diagnostic performance evaluation included the determination of the sensitivity and specificity of correct sample categorization at the boundaries of adjacent categories: Negative to indeterminate and positive to indeterminate. The sensitivity and 1-specificity were calculated and plotted as shown above. In addition, the Youden index was calculated and used to determine the cutoffs. The ROC assessment method followed the guidance document: CLSI EP24-A2: Assessment of Diagnostic Accuracy of Laboratory Tests Using Receiver Operating Characteristic Curves Approved Guideline-Second Edition. The sensitivity and specificity for the discrimination of negatives and positives were 94% and 75%, respectively.

The Fungitell STAT™ -derived BGI is designed to reflect the Fungitell^®^ predicate kit results interpretation, with some zone boundary changes. The BGI values reflecting the boundaries of the positive, negative, and indeterminate zones in the Fungitell STAT™ assay are BGI ≤0.74, negative; BGI ≥1.2, positive; BGI 0.75 to 1.1, indeterminate. These cutoffs were determined utilizing a ROC curve analysis of 93 patient samples tested with both methods providing categorical data to compare with the Fungitell^®^ predicate data. The boundary index values were determined from the Youden indexes (Table 2) of the ROCs presented in Figure 4 below. Table 3 compares the cutoff and range characteristics of Fungitell^®^ and Fungitell STAT™ data.

**Table 1.** Example of use of the Fungitell^®^ STAT standard to calculate the ratio or index.

Categorical examples	405–495 slope (OD/s)	405–495 intercept	405–495 *r*-value	Ratio/index
**A Positive**	0.000334	−0.3042	0.9998	2.21
**B Indeterminate**	0.000165	−0.1558	0.9994	1.09
**C Fungitell STAT™ standard**	0.000151	−0.1429	0.9994	1.00
**D Indeterminate**	0.000120	−0.1100	0.9989	0.79
**E Negative**	0.000069	−0.0595	0.9938	0.46

This patient sample index corresponds to either a negative, indeterminate, or positive result according to the clinical reference index ranges (see Table 3). The slope, intercept and *r*-values are evaluated as part of the general sample QC evaluation. Details provided with QC criteria in Supplementary File 1. These data are from Figure 1.

**Table 2.** Receiver-operator curve results and Youden indexes.

Positive cutoff							
Zone	ROC AUC	AUC 95% CI	AUC P (AUC = 0.5)	Cutoff value [rounded]	Sensitivity^2^ (%)	Sensitivity 95% CI	Youden index
**Positive** **^1^**	0.818	0.677, 0.959	<0.0001	>1.076 [>1.1]	75.0	62.2, 87.8	0.550

Negative cutoff							
Zone	ROC AUC	AUC 95% CI	AUC P (AUC = 0.5)	Cutoff value [rounded]	Specificity^3^ (%)	Specificity 95% CI	Youden index
**Negative** **^1^**	0.929	0.856, 1.000	<0.0001	≤0.751 [≤0.75]	94.1	86.2, 102.0	0.808

1 ROC for negative zone used negatives vs. indeterminates; ROC for positive zone used positives vs. indeterminates. AUC = area under the curve; CI = confidence interval.

2 Sensitivity for detecting pos in positive zone.

3 Specificity for detecting neg in negative zone.

**Table 3.** Comparative characteristics of Fungitell^®^ and Fungitell STAT™.

	Fungitell^®^ predicate (pg/mL)	Fungitell STAT™ (BGI^1^)
**Comparable overall range**	31–500	0.4–3.5
**Negative cutoff**	<60	≤0.74
**Indeterminate cutoff**	60–79	0.75–1.1
**Positive cutoff**	≥80	≥1.2

1 BGI = beta glucan index.

### Method comparison study: patient samples

An in-depth method comparison study confirming the validity of the cutoffs presented above was executed. In this study, a total of 488 samples were tested using both the predicate and Fungitell STAT™ methods. In Table 4 below, data were arrayed in a two-way table, comparing the positive, negative, and indeterminate status of all samples (based upon the predicate device reference results). These samples were analyzed for positive and negative percent categorical agreement between the methods, and the results are provided in Table 5. PPA with and without the indeterminate samples (by the predicate test, were 74% and 99%, respectively. NPA with and without the indeterminate samples (by the predicate test, were 91% and 98%, respectively). Thus, the ability of the Fungitell STAT™ assay to discriminate negatives from positives in the presence or absence of the Fungitell^®^ predicate indeterminate samples was very good and excellent, respectively. The accuracy of the Fungitell STAT™ in discriminating the Fungitell^®^ predicate positives in the absence of the indeterminate samples was also high but marginally lower with the indeterminates included. The lower rate of concordant positives by the Fungitell STAT™ assay was influenced by the lower positive cutoff of the predicate, relative to that calculated for Fungitell STAT™.

**Table 4.** Categorical results from Fungitell^®^ and Fungitell STAT™.

		Fungitell^®^			
		Negative	Indeterminate	Positive	Total
**Fungitell STAT™ ratio**	**Negative**	283	17	1	301 (61.7%)
	**Indeterminate**	19	17	24	60 (12.3%)
	**Positive**	7	2	118	127 (26.0%)
	**Total**	309 (63.3%)	36 (7.4%)	143 (29.3%)	488 (100%)

**Table 5.** Positive percent agreement (PPA) and negative percent agreement (NPA) and for Fungitell STAT™ compared to Fungitell^®^ predicate.

Method	Variable	Categorical results STAT/Fungitell	Fungitell STAT™ % concordance to Fungitell^®^	95% CI
Indeterminates not included	PPA	118/119	99%	95.4, 99.9
	NPA	283/290	98%	95.4, 99.9
Indeterminates included*	PPA	118/160	74%	66.4, 80.0
	NPA	283/311	91%	87.3, 93.7

The PPA and NPA were calculated with and without indeterminate values included. CI = confidence interval.

*If indeterminate results are considered discordant results (e.g., false positive or false negative) the calculations are as presented in Table 5.

### Reproducibility studies: interlaboratories

**Figure 5.**
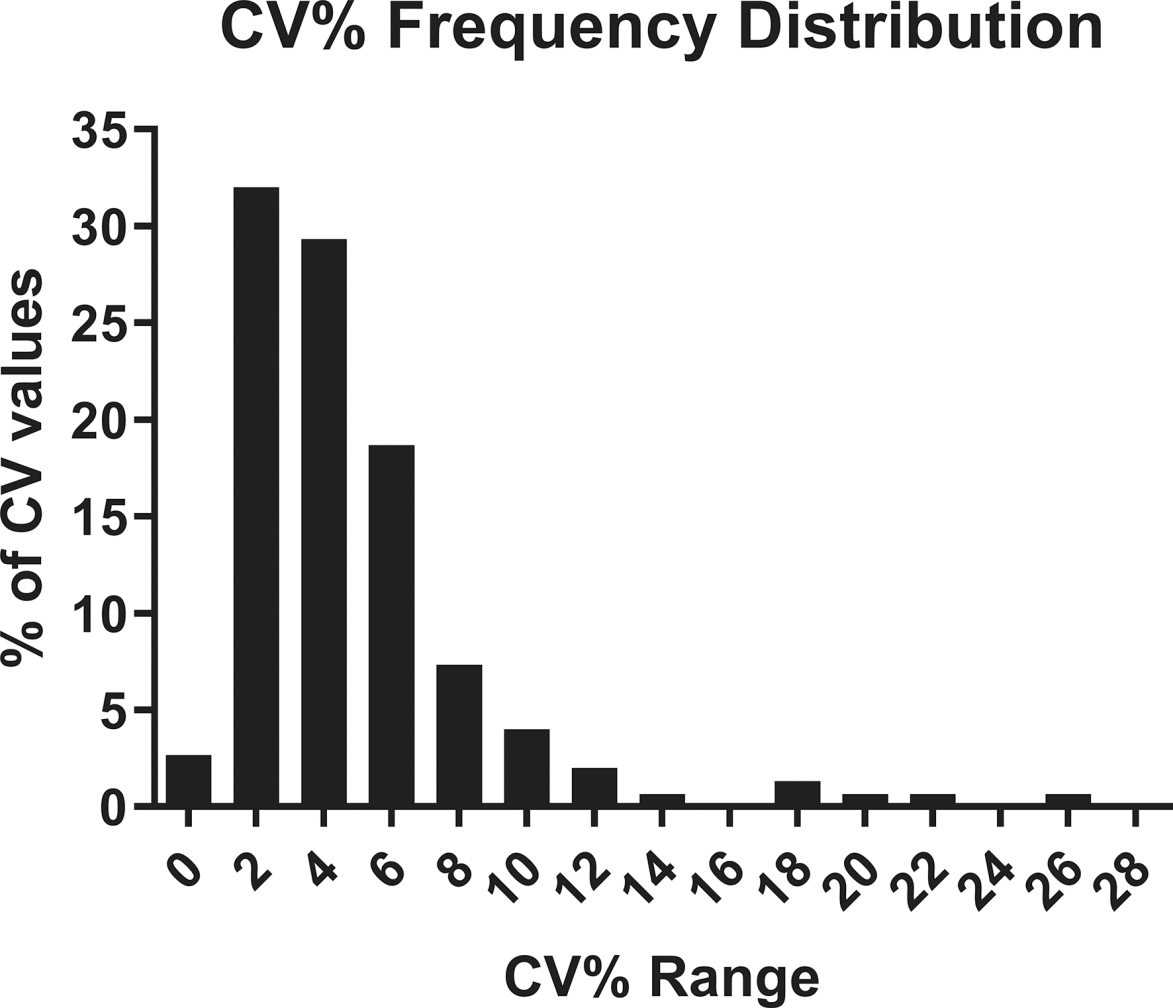
Intralab %CV frequency distribution for samples tested in the inter-lab study. This histogram contains 150 observations and *n* = 3 per observation for each of five samples provided to each lab (*n* = 3 per replicate analysis). The tests were conducted in three different labs over 5 non consecutive days by six different analysts using six different instruments. The distribution range was 0.5 to 27% with a mean of 4.9%.

Fungitell STAT™ was evaluated for precision/reproducibility by spiking human serum with *Saccharomyces cerevisiae* (1→3)-β-D-Glucan to produce a panel consisting of a low negative sample, high negative sample (just below the lower cutoff of 0.74), indeterminate (equivocal) sample, low positive sample (just above the upper cutoff of 1.2), and high positive sample (∼2× above the upper cutoff of 1.2). This panel was tested twice per day, in triplicate, at three sites by multiple operators over a 5-day period (1 panel member × 2 per day × 3 replicates × 3 sites × 5 days = 90 measurements per panel member) to determine the precision/reproducibility of the assay. Results are shown in Table 6. These five samples ranging in concentration from 34 to 233 pg/mL based on the Fungitell^®^ predicate were tested in three labs. The percent positive (% positive) represents the number of Fungitell STAT™ index values that fell within the positive zone. Several measures of precision were analyzed. In Figure 5 we present the intralab (*n* = 3) precision of repeat sample testing, demonstrating that 94% of % CV values were 10% or less. Accordingly, the results showed high reproducibility across multiple technicians in multiple laboratories with multiple instruments. Thus, the Fungitell STAT™ precision (intra-assay variation) % CV ranged from 0.5% to 27% and the interassay variation ranged from 11% to 20.4%. These % CV values are consistent with what was observed for the predicate Fungitell^®^.

**Table 6.** Interlab reproducibility study results.

Panel member	Mean index	Std. Dev.	% CV	% positive (no. positive/ no. tested)
Low negative	0.55	0.10	20.4	1.1% (1/90)
High negative	0.75	0.08	11.1	0.0% (0/90)
Indeterminate	0.94	0.10	11.1	3.3% (3/90)
Low positive	1.6	0.30	18.7	96.7% (87/90)
High positive	2.6	0.40	15.4	100% (90/90)

Combined data sets from three test sites included in this table. CV = coefficient of variation.

### Assay workflow duration

Once trained in the execution of the Fungitell STAT™ assay, the average hands-on time required for the preparation and pre-incubation of a standard and one or two samples through insertion into the instrument to start the kinetic assay was 30 minutes. The maximum time observed was 58 minutes and minimum time observed was 19 minutes. The kinetic run time is 40 minutes. Thus, it is possible on average to perform the sample preparation through kinetic assay commencement and complete data collection for a sample in 70 minutes.

## Discussion

Good discriminant performance and rapid turnaround time are key requirements of tests in infectious disease settings where early, appropriate therapy is critical to achieving successful outcomes.^22^ This is the case with invasive fungal disease (IFD), which is characterized by a rapid rise in morbidity and mortality when appropriate therapy is delayed by mere hours.^4–6^ Faced with such challenges, prophylaxis and empirical therapy is widely practiced and has led to high rates of administration of antifungals, based upon at-risk patient status or IFD being in the differential diagnosis.^23,24^ In such circumstances, there is a pressing need for diagnostic tools that can support safe antifungal drug stewardship practices.

Serum (1→3)-β-D-glucan (BDG) testing has been shown to provide both high sensitivity and excellent negative predictive value.^25^ Patient surveillance is optimal with testing performed 2–3 times per week with rapid result reporting. Fungitell^®^, the most widely utilized of the BDG tests exists in a microplate-based assay, which permits the testing of up to 21 samples per plate. This format is most suitable for large hospitals and reference labs where high sample throughput exists on a daily basis. Due to both logistic and economic reasons, testing tends to be underperformed in small sample number contexts and in emergent care. Delays incurred while samples are sent to reference labs or aggregated for batch testing increase turn-around-time (TAT) to undesirable levels. To address these issues, the Fungitell^®^ product line has been extended to include a single test design permitting single patient testing with a laboratory work period of approximately an hour (Fungitell STAT™).

Like the predicate device, the new format utilizes the measurement of the rate of para-nitroaniline (pNA) release due to hydrolysis by activated BDG-sensitive protease zymogens of the LAL-based reagent. Instead of interpolating sample BDG titers against a standard curve, the slope of the sample is divided by the slope of a simultaneously run Fungitell STAT Standard with a BDG titer at the positive threshold of the predicate device. This produces a (1→3)-β-D-glucan index value, or BGI, which was shown to vary linearly with the BDG titer in the sample, over the range of the predicate device (Fig. 3).

The performance of the new test format was evaluated against the predicate assay for a variety of factors. These included linearity of response over the range of the predicate (Fig.  2,  3), agreement relative to the three interpretative regions (Table 5), negative, indeterminate, and positive, and intra- and interlaboratory reproducibility (Fig. 2, 5). The assessment of the BGI method against the predicate's pg/mL values using patient samples resulted in an *r*-value of 0.92 (Fig. 3). Possible reasons for the deviations from an exact match between methods include data collection methods and analysis protocols used in the calculation of the slopes as well as the minor variances associated with the % CV of both methods. Despite these potential limitations, the STAT test, designed as a qualitative, categorical assay produced highly concordant negative and positive categorical agreement with the predicate assay (Table 5).

### BGI cutoffs

The evaluation of categorical agreement was established by testing 93 patient samples, previously tested by the predicate method, using two technicians and two instruments. The data were analyzed by the ROC methods, and the boundary determinations between negative and indeterminate and positive and indeterminate were selected using the Youden Index derived from the ROC output. These were 0.75 and 1.1 (Fig. 4), respectively. While the negative threshold corresponds almost exactly to the 60 pg/mL value of the predicate test, the positive threshold of the BGI corresponds to a slightly higher value at 1.1. In addition these ROC derived cutoff values were used as the lower and upper limits of the equivocal zone with the negative cut-off at ≤0.74 and the positive cutoff of ≥1.2. The use of these cutoffs at the limits of the indeterminate zone reflects in part the combination of the variability of both the Fungitell^®^ Predicate and Fungitell STAT™. It is noted that the BGI positive cutoff is higher than that predicted for the predicate level of 80 pg/mL, which would correspond to a BGI of 1.0. The offset of the positive cutoff in the STAT BGI test in this study reflects variability derived from the predicate assay, the BGI assay, and the patient sample set utilized. Given that the %CV averages for the interlab assay results are 11.7% and 4.9% for the predicate and Fungitell STAT™, respectively, with similar distributions, it is reasonable that there is some combined variance impact. The utility of these BGI values as negative and positive cutoffs, respectively, were confirmed in a larger patient sample set (Table 5).

### Percent positive and negative agreement

Four hundred and eighty-eight (488) patient samples with BDG titers across the range of the standard curve of the predicate device were tested. The results were analyzed for PPA and NPA. PPA with the inclusion of indeterminates as negative values was 74% and without the indeterminate values was 99%. NPA was 91% and 98% with and without indeterminates, respectively. Accordingly, the BGI method, leaving out the indeterminates, very accurately mimics the results of the predicate assay. The use of a higher BGI threshold, one that corresponds to approximately 96 pg/mL, affects the agreement between the PPA of the two methods, as BGI values between 1 and 1.2 do not contribute positives in the scoring of agreement with predicate values between 80 and 96 pg/mL. The slightly higher threshold of positivity for the BGI-based assay should result in higher specificity at a slight cost of sensitivity.

### Reproducibility

Three labs analyzed five samples that ranged between 34 and 233 pg/mL. Two technicians in each lab performed the assay, and two instruments were used. The sample sets were analyzed five times in triplicate; results indicated that 94% of the CV values were ≤10% and the average was approximately 5%. Accordingly, the BGI method was shown to have a suitable level of precision for the intended purpose.

### Workflow and elapsed time to result

The turn-around time (TAT) is a key driver in the laboratory support of IFD diagnosis. The time required to process the samples in the BGI method is an important characteristic. Using data obtained in the interlaboratory studies, it was observed that technicians with demonstrated proficiency required an average of 13 minutes of hands-on time and 30 minutes of elapsed time to begin collecting kinetic data. With a data collection period of 40 minutes, the time to obtain a result is approximately 70 minutes. Allowing for sample collection and transport, serum preparation, assay completion, and report preparation, it is entirely feasible for a hospital laboratory to report results in a timely manner. This compares favorably with the multiday TAT associated with batched samples and send-outs.

### Limitations

The work presented herein is a combination of both in-house and external laboratory consulting work. Fully independent assessment of the Fungitell STAT™ method is in progress and will be published when complete.

In summary, we have demonstrated that the Fungitell STAT™ BGI method permits a rapid, accurate, and reproducible (1→3)-β-D-glucan test to be implemented in low sample number or single patient emergent care contexts. Additionally, the BGI method is traceable to the large and growing body of data and experience with the established Fungitell^®^ predicate method.

## Supplementary Material

myaa028_Supplemental_File
