# Supplementary material for: Performance characteristics of Fungitell STAT™, a rapid (1→3)-β-D-glucan single patient sample in vitro diagnostic assay
Source: Med Mycol. 2020 May 13;59(1):41–9. doi: 10.1093/mmy/myaa028 (PMC7779209; doi:10.1093/mmy/myaa028)
Supplement: myaa028_Supplemental_File [file myaa028_supplemental_file.docx]

**Supplemental File 1: Quality Control Questions extracted from the Instructions for use for Fungitell STAT Standard and Sample Result Evaluation**

| **A.** | **Determine if Fungitell STAT Standard result is valid** |
| --- | --- |
|  | -the correlation coefficient (r) must be  ≥ 0.980 and  -the slope must be within the expected range |
|  | **Note:***If the Fungitell STAT Standard result does not meet criteria #1 and #2, the run is invalid and all samples should be run again.* |
|  |  |
| **B.** | **Determine if the sample is out-of-range** |
| **1** | **The result is likely out-of-range on the positive side:**  -If the Y intercept is positive and  -the kinetic curve passes 0.4 OD before 1000 seconds |
| **2** | **The result is likely out-of-range on the negative side:**  -If the kinetic curve is positive after 500 seconds and  -has an OD >0.03 and <0.07 at the end of the test |
|  | **Note:** *If the Sample result meets both criteria for either the positive or negative out-of-range, the basic QC criteria below do not need to be completed and the index values should* ***not*** *be calculated. All out of range results on the positive side should be reported at “Positive” and all out of range results on the negative side should be reported as ”Negative”.* |
|  |  |
| **C.** | **If the result does not meet the out-of-range criteria, verify the general QC:** |
| **1** | The kinetic curve must be positive after 500 seconds : Pass / Fail |
| **2** | The kinetic curve must have an OD ≥0.03 at the end of the test : Yes / No |
| **3** | The slope must be numerically positive: Pass / Fail |
| **4** | The r-value is ≥ 0.980: Pass / Fail |
| **5** | The kinetic curve must have an upward increasing curve shape consistent with examples: Pass / Fail |
|  | **Note:** *If the Sample result does not meet all general QC criteria #1-5, the sample result is invalid and the sample has to be tested again.* |
